# Noncoding RNAs Carried by Extracellular Vesicles in Endocrine Diseases

**DOI:** 10.1155/2018/4302096

**Published:** 2018-04-01

**Authors:** Margherita A. C. Pomatto, Chiara Gai, Maria Chiara Deregibus, Ciro Tetta, Giovanni Camussi

**Affiliations:** ^1^Stem Cell Laboratory, Department of Medical Sciences, University of Turin, Turin, Italy; ^2^2i3T Scarl, Univerity of Turin, Turin, Italy; ^3^Unicyte AG, Oberdorf, Nidwalden, Switzerland

## Abstract

RNA molecules are essential and fine regulators of important biological processes. Their role is well documented also in the endocrine system, both in physiological and pathological conditions. Increasing interest is arising about the function and the importance of noncoding RNAs shuttled by extracellular vesicles (EVs). In fact, EV membrane protects nucleic acids from enzyme degradation. Nowadays, the research on EVs and their cargoes, as well as their biological functions, faces the lack of standardization in EV purification. Here, the main techniques for EV isolation are discussed and compared for their advantages and vulnerabilities. Despite the possible discrepancy due to methodological variability, EVs and their RNA content are reported to be key mediators of intercellular communication in pathologies of main endocrine organs, including the pancreas, thyroid, and reproductive system. In particular, the present work describes the role of RNAs contained in EVs in pathogenesis and progression of several metabolic dysfunctions, including obesity and diabetes, and their related manifestations. Their importance in the establishment and progression of thyroid autoimmunity disorders and complicated pregnancy is also discussed. Preliminary studies highlight the attractive possibility to use RNAs contained in EVs as biomarkers suggesting their exploitation for new diagnostic approaches in endocrinology.

## 1. Introduction

The larger fraction of transcribed RNAs is composed by noncoding RNAs, instead of mRNAs coding for proteins [1]. Noncoding RNAs include a wide variety of RNA molecules, such as transfer RNAs (tRNAs), ribosomal RNAs (rRNAs), small nuclear RNAs (snRNAs), circular RNAs (cRNAs), and small nucleolar RNAs (snoRNAs) with several different regulatory and structural functions. They are involved in mRNA translation and splicing, transcription initiation, but also cell cycle regulation, chromosome maintenance and segregation, chromatin remodeling, and epigenetic memory regulation [1]. Noncoding RNAs also include cytoplasmic Y RNAs (yRNAs) and vault RNAs (vtRNAs). YRNAs are involved in chromosomal DNA replication and in RNA stability when complexed with Ro60 ribonucleoprotein particle. YRNAs also modulate cell death and inflammation [2, 3]. VtRNAs are a part of large ribonucleoprotein particles present in the cytoplasm of many eukaryotic cells, which are suggested to be involved in several processes, including multidrug resistance of cancer cells, DNA damage repair, innate immune response, apoptosis resistance, nuclear pore complex formation, and nucleocytoplasmic transport [4, 5]. However, their functions are still not completely elucidated. Two other well-studied classes of noncoding RNAs are microRNAs (miRNAs) and small interference RNA (siRNAs), short single-strand RNA molecules (20–22 nucleotides) derived from hairpin or double-stranded RNA precursors. These RNAs are loaded by the Dicer complex into a member of the Argonaute protein subfamily to form the RNA-induced silencing (RISC) complex, which recognizes a complementary sequence in the target mRNA and mediates degradation or inhibits translation into protein [6]. miRNAs regulate posttranscriptional gene silencing of up to 60% of protein-coding genes targeting one or several mRNAs. They have been associated to quite all biological processes, including development, proliferation, differentiation, metabolism, apoptosis, and cancer [7]. Finally, a large part of the mammalian noncoding transcripts is composed by long noncoding RNAs (lncRNAs), RNA molecules of approximately 200 nucleotides or more. LncRNAs take part in several biological processes: they regulate transcription by affecting the activity of specific transcription factors and polymerases. They mediate posttranscriptional regulation, by influencing splicing, transport, translation, and degradation of mRNAs, and they take part in epigenetic modifications, by regulating chromatin remodeling and X chromosome inactivation in mammals [8, 9]. Interestingly, lncRNAs can also modulate the biological activity of other RNA species. LncRNAs can interact with miRNAs and inhibit their effect by acting as “competing endogenous RNAs” (ceRNAs). LncRNAs containing miRNA-binding sequence regions can impound miRNA molecules and reduce their availability for target mRNAs [10]. This mechanism of interaction has been recently demonstrated to play a critical role in several pathological processes, including cancers [11–13], fat deposition [14], diabetic retinopathy [10], and osteoarthritis [15], and in biological processes such as cellular apoptosis [16–18] and stem cell differentiation [19].

During the last few years, extracellular vesicles (EVs) have been recognized as carriers of RNA molecules from their cell of origin to recipient cells all over the organism. Indeed, EVs are a heterogeneous class of vesicles ranging from 20 to 1000 nm, delimited by plasma membrane (PM) and containing proteins and nucleic acids. In this review, we will collectively refer to EVs that include several subpopulations, such as microvesicles, microparticles, and exosomes, basing on their size, biogenesis, molecular markers, and isolation techniques [20, 21].

## 2. EVs and Communication

It is now clear that EVs play an important role in cell to cell communication between neighboring and distant cells. In fact, EVs are released by quite all cell types and have been detected in several biological fluids [22]. Recipient cells uptake EVs by receptor-mediated interactions or by direct fusion with the PM [23]. In this way, EVs transfer lipids, membrane receptors, proteins, or nucleic acids to recipient cells. First evidence that EVs mediate the horizontal transfer of proteins and mRNAs and reprogram recipient cells was provided by Ratajczak et al. [24]. Recently, some interesting *in vivo* studies have used Cre recombinase technique [25–27] or a combination of fluorescent and bioluminescent reporters tagging EV membrane and RNA molecules [28] to demonstrate that mRNAs are transferred from cell to cell by EVs. Being mediators of cell to cell interaction, EVs play a complex role in pathophysiology of several organs and diseases. The biological effects of EVs depend on their origin, on the status, and on the environment. Stem cell-derived EVs elicit, at least in part, the regenerative properties of their cell of origin. Particularly, EVs released by mesenchymal stromal cells (MSCs) have been widely studied and showed immunomodulatory properties and a protective role in cardiovascular disease, kidney disease, lung disease, and liver disease [29]. MSC-derived EVs exploit both antitumor and protumorigenic activity based on the context [30]. On the other hand, EVs released by cancer stem cells, such as renal cancer stem cells [31] or prostate cancer stem cells [32], can promote tumor metastasis. In general, EVs act influencing different biological processes and tumor-derived EVs can support tumor in several ways, by promoting cell migration, invasiveness, angiogenesis, and premetastatic niche formation in distant sites [33]. EVs from different other cells, adipose mesenchymal stem cells (ASCs), endothelial cells, proangiogenic progenitors, and tumor cells, show proangiogenic properties *in vitro* and *in vivo*, in ischemic injury or in tumors [34].

## 3. EV Biogenesis and Cargo

EVs can be classified into two different groups based on their biogenesis: microvesicles and exosomes. Microvesicles originate by shedding from the cell surface. Changes in the composition of lipids, proteins, and other components of the PM modify the curvature of the membrane and facilitate the microvesicle budding. This process relies, in part, on the interaction of proteins such as arrestin domain-containing protein-1 (ARRDC1) with the late endosomal protein TSG101. Microvesicle fission is due to myosin and actin cytoskeletal rearrangements regulated by the Ras-related GTPase ADP-ribosylation factor 6 (ARF6) and its signaling cascade [35]. Exosomes are formed by budding of the membrane into the lumen of multivesicular bodies (MVB), which are part of the endocytic pathway and fuse with the PM releasing exosomes outside the cell. Several proteins are involved with the formation of exosomes, such as the components of the endosomal sorting complex required for transport (ESCRT) machinery and the accessory proteins TSG101, ALIX, and VPS4. However, other ESCRT-independent mechanisms have been described [36]. The docking of MVB with the PM is mediated by several components of the RAB family of small GTPase proteins (RAB2B, RAB5A, RAB7, RAB9A, RAB11, RAB27A, RAB27B, RAB35 [37], and RLP-1 [38]). Some members of the tetraspanin family, such as CD63, CD81, and CD9, are enriched in exosomes and have been recognized as exosome markers [37]. On the other hand, specific markers for microvesicles are lacking and it is difficult to discriminate EVs based on biomarkers. Moreover, even if the biogenesis of microvesicles and exosomes is based on different processes, some mechanisms are shared [36].

EV content varies depending on the originating cell and the biogenesis mechanism. However, the compartmentalization of proteins and RNAs is, at least in part, a regulated process. Comparative lipidomic, proteomic, and transcriptomic analysis usually finds an enrichment of subsets of lipids, proteins, or RNAs in EVs compared with their cells of origin [39–43]. Several proteins involved in EV biogenesis regulate compartmentalization into EVs. For example, ESCRT complex recruits proteins into both exosomes and microvesicles [44]. In human liver stem cells, ALIX is associated with Ago2, a member of the Argonaute protein family, which binds miRNAs. The complex ALIX-Ago2-miRNA was found in EVs [45]. In breast cancer, EVs contain functional Ago2-associated miRNAs, which are mature and induce transcriptome alterations in target cells [46]. The heterogeneous nuclear ribonucleoprotein A2B1 (hnRNPA2B1) recognizes specific motifs and regulates miRNA loading into EVs [47]. The RNA-binding protein Y-box protein I (YBX1) binds to miR-223 and is necessary for its packaging into EVs [48]. In colorectal cancer cells, KRAS seems to mediate miRNA sorting into EVs [49] by regulating Ago2 secretion [50]. Besides miRNAs, increasing reports show that EVs are particularly enriched in other types of small RNAs, such as tRNA fragments, yRNAs, vtRNAs, and miRNA fragments [51–53]. The presence of a regulated mechanism of small RNA sorting into EVs has been observed also in Leishmania, suggesting that it is conserved throughout evolution [54]. However, the biological functions of small RNAs found in EVs are not completely clear.

## 4. Techniques for EV Isolation

Before addressing the main topic of this review relative to the role of noncoding RNA shuttled by EVs in endocrine pathophysiology, it is useful to discuss the technical challenges in EV purification as these may influence the analysis of RNAs contained in EVs [55]. In fact, EVs are heterogeneous in size and molecular composition (membrane lipids, surface proteins, and cargo), and thus in density and charge. The heterogeneity is increased as EVs derive from different cell of origin (e.g., biological fluids such as blood or saliva). This is a relevant problem since the purity, the integrity, the yield, and the biological activity of EVs are influenced by the isolation technique [56, 57]. Each isolation technique presents advantages and disadvantages, and the choice of the methods should be based on the starting material and volume, on the grade of purity desired, and on the purpose of the isolation (research, therapeutic, or diagnostic use).

The standard technique is differential ultracentrifugation. This protocol is based on several consecutive centrifugation steps. First steps are necessary to discard cells and debris and consist in brief centrifugation at low centrifugal forces (*g*). The consecutive steps isolate different populations of EVs based on density (i.e., 10,000 g for microvesicles and 100,000 g for exosomes). The protocol was firstly described by Raposo et al. [58] and then widely adopted with slight variations depending on the source of EVs (cell supernatant or biological fluids) [59, 60]. It is often combined with other techniques, mostly the density gradient with or without cushion, to stratify EVs based on their density and obtain a pure population, free of contaminant proteins, especially to perform proteomic or other molecular analysis [61–64]. Iodixanol (OptiPrep), rather than sucrose, is preferred for gradient because it reduces contaminants such as HDL [65, 66], small apoptotic bodies, and virus [67]. Recently, an upward floating method into iodixanol gradient has been proposed by Kowal et al. [68] to better separate and to characterize subtypes of EVs. Some concerns exist over the possibility that high-speed centrifugation may damage EVs and create aggregates [69]. Another issue is related to the low reproducibility of the technique. Adopting different rotors, speeds, and times, yield, protein, and RNA content of the pellet might vary [70]. Nevertheless, differential ultracentrifugation is still the most widely used method, especially for conditioned cell culture media [64].

Another technique is size-exclusion chromatography (SEC). EVs flow into a filtration column that elutes EVs of different sizes in different fractions. This method is preceded by a preconcentration step and followed by ultracentrifugation that can damage EVs [71]. In other cases, SEC is used as a stand-alone technique [72, 73]. It seems that SEC is preferentially used to isolate EVs from complex biological fluids, such as plasma [74, 75], urine [76], and milk [73]. Two studies comparing ultracentrifugation and SEC suggest that SEC provides a higher yield of EVs [77] and contaminant reduction [78]. Other size-based isolation methods are ultrafiltration, flow field-flow fractionation, hydrostatic filtration dialysis [79], an integrated double-filtration microfluidic device [80], and lab-on-a-disc integrated with two nanofilter devices [81].

Moreover, EVs can be isolated with immunoaffinity capture-based techniques, which use microbeads coated with antibodies for EV membrane receptors to recognize and isolate EVs. This approach selects specific subpopulations of EVs without considering their size or density [71]. Therefore, this method may be unsuitable for some application, but it is very useful for others, such as the detection of diagnostic or prognostic markers expressed on EV surfaces. Moreover, this method can be coupled to flow cytometry, Western blotting, and real-time PCR to furtherly characterize EVs [71]. The same method has been used to develop an ELISA microplate for the capture and quantification of EVs from urine, serum, and plasma [82]. Besides, immunoaffinity isolation is more used for complex biological fluids than for cell supernatants, or with small starting volumes [64].

Another method of EV isolation is based on precipitation. Polymers such as polyethylene glycol (PEG), which separate water from solutes, are incubated overnight at 4°C with cell culture media or biological fluids, and EVs are collected by low-speed centrifugation or filtration [83]. Moreover, EV negative charge has been exploited to develop a charge-based precipitation method, which uses protamine, a positively charged molecule, coupled with PEG to separate EVs [84]. Precipitation techniques recover EVs more efficiently than ultracentrifugation [84, 85]. Moreover, precipitation techniques require very small volumes and are easier to perform. For this reason, this technique is suitable for diagnostic use. On the other hand, it is possible to coprecipitate contaminants, such as lipoproteins and the polymer itself [86]. However, these particles can be removed by a preisolation centrifugation and a post isolation filtration through Sephadex G-25 spin columns [57, 84].

## 5. Metabolic Syndrome

In the last years, it has been suggested that miRNAs, both intracellular and extracellular, can control the metabolic homeostasis and may impact the main tissues involved in the development of the metabolic syndrome [87]. In effect, miRNAs represent important modulators of glucose and lipid metabolism in the liver [88], insulin production in the pancreas [89], and leptin signaling in the hypothalamus [90]. In addition, miRNAs have been involved in many processes associated to metabolic disorders such as oxidative stress, inflammation, insulin signaling, adipogenesis, and angiogenesis. In this part, we describe the recent knowledge on the role of RNAs shuttled by EVs in metabolic syndrome and correlated disorders. Most relevant EV-cargoes involved in endocrine diseases discussed here and in the following paragraphs are summarized in Table 1.

### 5.1. Obesity

Obesity is a chronic condition characterized by an excessive accumulation of body fat potentially associated to other metabolic disorders, hypertension, insulin resistance, and cardiovascular diseases. Human obesity associates with a chronic state of low-grade inflammation with augmentation of inflammatory protein production, increased infiltration of macrophages, lipolytic activity of adipocytes with increased systemic liberation of fatty acids, and potential insulin resistance. The increased production of proinflammatory cytokines and the decrease of anti-inflammatory cytokines may take part in the metabolic complications linked with obesity and leading to metabolic syndrome. This condition is modulated by signal transduction networks, and miRNAs may regulate the expression and production of inflammatory proteins with amplification of the inflammatory effect [91]. Adipose tissue is a heterogeneous tissue composed by several cell types. Adipocytes and ASCs for instance may release EVs that regulate adipocyte metabolism. In effect, when the adipose-specific knockout of Dicer is performed, a depletion of most miRNAs is observed [92–94]. miRNA deficit prejudices adipocyte basic functions like differentiation, metabolism, and signaling [94]. It has been shown that several miRNAs may contribute to the infiltration of macrophages in the adipose tissue [95–97] and to their transition to a proinflammatory phenotype [98–101] favoring the low-grade chronic inflammation characteristic of obese people and the induction of insulin resistance [102].

A recent work by Ying et al. [103] showed that adipose tissue macrophages may regulate systemic insulin responses via EVs. The authors reported that macrophages from adipose tissue of obese mice released EVs carrying miRNAs able to induce insulin resistance and glucose intolerance in lean mice. At variance, EVs from adipose tissue macrophages of lean mice were able to ameliorate glucose metabolism and insulin sensitivity of obese mice. MiR-155, targeting the peroxisome proliferator-activated receptor gamma (PPAR*γ*) gene, was upregulated in EVs of obese adipose tissue macrophages. EV-mediated transfer of miR-155 to insulin target cells of the liver and muscle may have an impact on metabolism [103]. In humans, PPAR*γ* exerts several important functions in adipocyte differentiation, lipid metabolism, and glucose homeostasis [104]. As a matter of fact, some studies have outlined that EVs released from adipose tissue may take part in the metabolic features associated with obesity [105–107] and differ from those of lean individuals [107, 108].

The potential benefits of EVs for biomarker identification are related to their easy purification, their stability when stored at −80°C, the protection of nucleic acids from enzyme degradation, and the possibility of EV subset discrimination based on the expression of membrane markers of the cell of origin. Karolina et al. [108] analyzed the profile of miRNAs in EVs of patients with metabolic syndrome, hypercholesterolemia, hypertension, or type 2 diabetes mellitus alone, showing a distinct miRNA profile for each single disease. Ferrante et al. [107] demonstrated a different expression of several miRNAs in obese in respect to lean visceral adipocyte EVs. In particular, downregulation of miR-148b and miR-4269 and upregulation of miR-23b and miR-4429 were reported in obese visceral adipocyte EVs. These altered miRNAs targeted the TGF-*β* and Wnt/*β*-catenin signaling linked to the modulation of chronic inflammation and adipogenic differentiation of ASCs [107]. It has been shown that EVs from adipose tissue intravenously injected in an obese mouse model (B6 ob/ob mice) or in mice fed with a high-fat diet were incorporated in peripheral blood monocytes following macrophage activation in a TLR4-dependent way. This led to increased secretion of TNF-*α* and interleukin-6 and development of insulin resistance [105]. Recently, Thomou et al. [109] demonstrated that the adipose tissue is a great source of circulating vesicular miRNAs capable of modulating gene expression in other tissues. The authors showed that circulating vesicular miRNAs are decreased in mice with a fat-specific knockout of Dicer, and in humans with lipodystrophy. The transplantation of white adipose tissue and mostly of brown adipose tissue into Dicer knockout mice was followed by restoration of circulating vesicular miRNAs, amelioration of glucose tolerance, and reduction of fibroblast growth factor-21 (FGF21) mRNA in the liver and of protein in blood [109]. FGF-21 is a hormone that is induced in the liver by fasting [110] and was repressed when liver cells were incubated with normal serum EVs but not with Dicer knockout EVs [108]. In EVs from Dicer knockout mice, the effect was restored by introduction of miR-99b, a predicted regulator of mouse FGF21. Moreover, the authors demonstrated that transplantation of brown adipose tissue reestablished other miRNAs such as miR-325 and miR-743b, predicted to target the uncoupling protein 1 (UCP-1, an uncoupling protein found in the mitochondria of brown adipose tissue), and miR-98 predicted to target the peroxisome proliferator-activated receptor gamma coactivator 1-alpha (PGCI*α*), the master regulator of mitochondrial biogenesis. Taken together, these data suggest that miRNAs carried by EVs could be transferred to other tissues where they are able to modulate gene expression [109]. It has been shown that ASCs release EVs capable of promoting endothelial cell migration, proliferation, and neoangiogenesis [111]. In obese subjects, the ASC-EV angiogenic potential resulted weakened in comparison with normal individuals with reduced endothelial cell migration and vessel-like structure formation [112]. This effect was mainly due to a reduced miR-126 content in EVs leading to Spred1 upregulation and inhibition of the extracellular signal-regulated kinase 1/2 mitogen-activated protein kinase (Erk1/2 MAPK) pathway in endothelial cells. Moreover, the treatment of nonobese ASCs with palmitic acid induced the release of EVs similar to those from obese, whereas the treatment of obese ASCs with high glucose decreased the miR-126 content in EVs and the *in vitro* angiogenesis [112].

Beside miRNAs, EVs were shown to contain lncRNAs [113]. Little is known on the role of lncRNAs carried by EVs in endocrine physiology and pathology. However, it has been reported that lncRNAs exert critical roles in homeostasis and differentiation of metabolic tissues interacting with target mRNAs or miRNAs [114, 115].

To date, accumulating evidence supports an association of diabetes and metabolic syndrome to liver disease [116, 117]. Like EVs released by adipose tissue, EVs produced by hepatocytes are sensitive to metabolic signals, but their importance in the interplay between metabolically active tissues and the liver is still uncertain [118]. It has been suggested that EVs carrying miRNAs released from different hepatic cells are involved in liver functions and play a role in liver diseases [119, 120].

Obesity and metabolic syndrome may enable the accumulation of fat in the hepatic tissues with the development of nonalcoholic fatty liver disease (NAFLD) [121]. It can initiate as simple steatosis, then advance to nonalcoholic steatohepatitis (NASH) in which inflammation and fibrosis associate to steatosis, eventually leading to cirrhosis [122]. It has been shown that macrophages and natural killer T cells of subjects suffering NAFLD or NASH release a higher amount of EVs in the circulation, in respect to healthy individuals [123]. Since their release correlated with alanine aminotransferase (ALT) levels and the degree of histologic damage, EVs have been suggested as a new noninvasive and quantitative diagnostic tool [123]. Povero et al. [124], in a NASH *in vitro* model of lipotoxicity, demonstrated that hepatocytes released EVs with proangiogenic activity on endothelial cells. This process was dependent on the EV membrane expression of the protein vanin-1, which mediated EV internalization in target cells and subsequent caspase 3 activation. Therefore, hepatocyte-derived vanin-1-positive EVs have been suggested as potential biomarker or target for therapy of NASH [124]. Moreover, the same authors showed that the quantity of liver and blood EVs was markedly increased in a choline-deficient L-amino acid (CDAA) diet experimental model of NAFLD in comparison with control mice [125]. The number of EVs increased over time and correlated with the degree of histopathological lesions. Interestingly, these circulating EVs showed a different pattern of proteins and an enrichment of two well-known miRNAs highly expressed in hepatocytes, miR-122 and miR-192. These results suggest a potential use of these EVs as biomarkers of hepatic damage [125]. Hirsova et al. [126] showed that toxic lipids, such as palmitate and its active metabolite lysophosphatidylcholine, induced an increased release of EVs from human hepatocytes. This process was mediated by the activation of the death receptor 5 signaling pathway in hepatocytes with downstream activation of caspases and of Rho-associated coiled-coil containing protein kinase 1 (ROCK1) [126]. These EVs carried the protein tumor necrosis factor-related apoptosis-inducing ligand (TRAIL) and could represent a possible connection between liver lipotoxicity and macrophage-mediated inflammation in NASH. In fact, the treatment with the ROCK1 inhibitor fasudil decreased the number of total and hepatocyte-derived circulating EVs in an *in vivo* model of NASH. Importantly, it induced a reduction of inflammation, hepatic damage, and fibrosis, suggesting the role of EVs in these mechanisms [126]. Moreover, Koeck et al. [127] isolated EVs from visceral adipose tissue of obese and lean patients and investigated their effects on cultured hepatic cell lines. Interestingly, incubation of liver cells with EVs from obese subjects dysregulated the TGF-*β* pathway, a process correlated with the pathogenesis of NAFLD [127]. As mentioned above, Ferrante et al. [107] demonstrated that EVs derived from obese subjects carry miRNAs which interfere with TGF-*β* pathway, suggesting their involvement in liver diseases. A recent study of Thomou and colleagues demonstrates that EVs derived from adipose tissue can guide metabolic insults in the liver via the transfer of miRNAs [109].

### 5.2. Diabetes

#### 5.2.1. EVs in Endocrine Pancreas Physiology

Pancreatic beta cells, situated within the islets of Langerhans, are crucial for the regulation of blood glucose homeostasis by secreting insulin. Several evidences demonstrated that noncoding RNAs control beta cell function and viability in health and disease [128]. Recently, it has been demonstrated that these cells release EVs involved in signal interactions influencing the activity of adjacent pancreatic beta cells. In fact, EVs isolated from beta cell lines and from rat, mice, or human islets contained miRNAs that can be transferred to neighboring beta cells, influencing their behavior. Importantly, EV-miRNA content varies based on cell conditions and EVs released by beta cells treated with proinflammatory cytokines were shown to lead to apoptosis of naive beta cells [129]. Besides the direct interaction between beta cells, the physiopathology of pancreatic islets has been demonstrated to depend on a crosstalk between beta cells and endothelial cells [130]. EVs seem to contribute to cell communication, and EVs isolated from human pancreatic islets were shown to be biologically active, inducing insulin mRNA expression, enhancement of angiogenesis, and protection from apoptosis. In fact, these EVs shuttle specific mRNAs and miRNAs into target human islet endothelial cells, including several mRNAs (VEGFa, eNOS) and microRNAs (miR-27b, miR-126, miR-130, and miR-296) involved in beta cell function, insulin secretion, and angiogenesis [131]. Moreover, EVs derived from endothelial progenitor cells (EPCs) induce *in vitro* islet endothelial cell migration, proliferation, organization in vessel-like structures, and resistance to apoptosis. *In vivo*, EVs were shown to favor survival, insulin secretion, and revascularization of islets transplanted in SCID mice. Of note, their effect is due to the transfer of proangiogenic miRNAs, miR-126 and miR-296 [132].

#### 5.2.2. EVs in Diabetes

Insufficient and/or ineffective insulin secretion or both of them can lead to diabetes mellitus (DM). The autoimmune beta cell destruction in the pancreas is the cause of insulin-dependent type 1 diabetes (T1D), whereas insulin resistance (IR) or inadequate insulin release is the cause of non-insulin-dependent diabetes (T2D). A potential involvement of miRNAs in the dialogue between immune system and pancreatic islets has been suggested [133]. A recent study [134] has suggested that EVs may take part in the beginning and acceleration of autoimmune pancreatic islet reactions in T1D demonstrating that rat and human pancreatic beta cells secrete EVs carrying autoantigens GAD65, IA-2, and insulin/proinsulin which target and activate dendritic cells promoting autoimmunity in particular under proinflammatory conditions. Evidence indicates the crucial role of miRNAs in the beta cell differentiation, acquirement of a mature phenotype, and dysfunction. In effect, the pancreatic-specific deletion in mouse embryos of Dicer was followed by a dramatic deficiency of insulin producing beta cells linked to upregulation of the notch-signaling target Hes1 and decrease in the formation of endocrine progenitor cells expressing the neurogenin3 gene [135].

In DM, miRNAs can regulate various molecular and cellular pathways like insulin synthesis and secretion in pancreatic beta cells, exocytosis of insulin granules, beta cell fate, and formation of islets [136]. Many miRNAs contribute to the adaptive features of beta cells to increase requirements of insulin such as miR-132 [137, 138], miR-184 [139], miR-338-3p [140], and miR-375 [141, 142].

EVs regulate intercellular signaling in diabetes mediating the exchange of RNAs between cells. Recently, miRNAs delivered by EVs have been demonstrated to mediate the crosstalk between skeletal muscle and beta cells in diabetes [143]. This study used mice fed with a high palmitate diet inducing hyperglycemia, glucose intolerance, hyperinsulinaemia, and insulin resistance. Interestingly, EVs isolated from skeletal muscle can be internalized by pancreatic beta cells with delivering of proteins and miRNAs, suggesting that they contribute to modification of the beta cell mass arising during insulin resistance. In particular, lipid-induced insulin-resistant muscle EVs transferred miR-16 to beta cells regulating Ptch1, a receptor involved in the sonic hedgehog pathway and suggested to exert a significant role in insulin resistance development [143].

In addition to miRNAs, EVs can shuttle other noncoding RNAs, which regulate cell gene expression also interacting with miRNAs. In fact, recent studies have shown that EVs may carry and transfer cRNAs that may have functional and diagnostic relevance [144, 145]. In particular, the human ciRS-7 (also termed Cdr1as) was detected in EVs [146] and is the first miRNA sponge identified, negatively regulating miR-7 [147]. Of note, a recent study underlined a critical relationship between ciRS-7/miR-7 and diabetes [148]. The authors demonstrated how the overexpression of Cdr1as augmented the insulin mRNA concentration and the granule secretion in beta cells via the inhibition of miR-7 in islet MIN6 cells and mouse islets [148]. In fact, miR-7 has been extensively related to diabetes impairing insulin secretion and beta cell dedifferentiation. Importantly, the knockdown of miR-7 in obese mice has been shown to ameliorate beta cell failure and glycemia [149]. Then, Cdr1as has been shown to improve beta cell function by preventing miR-7 interaction with its target genes. Among these, the authors demonstrated the importance of the paired box protein Pax-6 (Pax-6), which enhances insulin gene 1 (Ins1) and gene 2 (Ins2) transcripts by directly binding to their promoters increasing insulin content and secretion, and of Myrip (myosin VIIA and Rab-interacting protein), which forms a complex with Rab27a and MyosinVa and mediates insulin granule transportation and secretion [148]. These data suggest the presence of multiple types of vesicular RNAs involved in diabetes and highlight the necessity of further investigations to understand their complex role in this pathology. Taken together, these results suggest the therapeutic potential of EV-RNAs to induce the rescue of beta cell function.

Finally, miRNAs have been suggested as potential biomarkers for both type 1 and type 2 diabetes [150]. Recently, Garcia-Contreras et al. [151] have shown that plasma-derived EVs express a distinct signature in long lasting diabetes. Several deregulated miRNAs have been shown to be involved in diabetes progression. Barutta et al. [152] studied urinary EVs in incipient diabetic nephropathy showing an enrichment of miR-130a and miR-145 and a reduction of miR-155 and miR-424 in patients with microalbuminuria. In an experimental model of diabetic nephropathy, a similar upregulation of miR-145 in urinary EVs as well as in glomeruli was observed [152]. *In vitro* experiments showed that high glucose exposure of mesangial cells upregulated miR-145 with consequent increased expression of this miRNA in secreted EVs [152]. Since tissue-specific EVs may allow monitoring of transplanted tissue rejection, EVs could be also used as biomarkers to monitor the outcome of islet transplantation [153]. Moreover, it has been recently suggested that EVs carrying small RNAs released from MSCs may act as immune modulators to improve islet transplantation [154]. In fact, MSC-derived EVs have been already demonstrated to modulate immunity in type 1 diabetes inducing the formation of regulatory dendritic cells [155, 156].

### 5.3. Atherosclerosis

Dysfunctional adipose tissue and proinflammatory signals of metabolic syndrome are associated to the development of atherosclerosis, a condition characterized by a low-grade chronic inflammation of arterial wall [157, 158]. The process is initiated by endothelial damage and followed by deposition of lipoproteins in the subendothelial place. Beside signaling and molecular regulatory pathways critical for the formation and evolution of the atherosclerotic plaques, miRNAs are considered crucial modulators at a fine tune level of the different players implicated in the pathophysiological processes of atherosclerosis. In response to different stimuli, miRNAs such as the miR-181 family are suggested to be capable of modifying the balance of pro- and anti-inflammatory molecules implicated in the pathogenesis of atherosclerosis. The miR-181 family has a crucial role in vascular inflammation by regulation of signaling pathways and targets known to be critical for endothelium activation and immunity, thus contributing to the onset and development of vascular inflammatory diseases [159]. Several miRNAs involved in lipid metabolism have emerged as contributors of the pathogenic process of atherosclerosis such as miR-33 [160, 161], miR-27a/b [162, 163], and miR-122 [164]. miRNAs mediating the endothelial damage induced by disturbed flow have been identified and extensively studied (see [165] for review). In situations of shear stress, it has been shown that the increasing of the Kruppel-like factor (KLF2) transcription factor induced the expression of functional miR-143/miR-145 cluster which was loaded in endothelial cell EVs and taken up by vascular smooth muscle cells in coculture [166], with acquisition of a contractile phenotype and stabilization of endothelium. Noteworthy, EVs from endothelial cells were able to decrease atherosclerotic lesion formation *in vivo* in the aorta of ApoE^−/−^ mice in a miR-143/miR-145-dependent manner exerting an atheroprotective effect [166]. In another study, the exchange of miR-143/miR-145 through intercellular tunneling nanotubes has been shown to occur between vascular smooth muscle cells and endothelial cells resulting also in this case in vessel stabilization [167]. EVs derived from vascular smooth muscle cells overexpressing Kruppel-like factor 5 (KLF5) contained miR-155 and were able to promote atherosclerotic progression *in vitro* and *in vivo*. Thus, EVs could either promote or prevent atherosclerosis based on their RNA content [168]. In a recent study, de Gonzalo-Calvo et al. [169] investigated the miRNA content in EVs released from human coronary artery smooth muscle cells (CASMC) and its modification after exposure to atherogenic lipoproteins. Interestingly, these miRNA modifications were reproduced in the plasma of patients with familial hypercholesterolemia (FH). Among miRNAs deregulated by atherogenic lipoproteins, plasma miR-130a was suggested as potential biomarker of significant coronary atherosclerosis, and miR-24-3p and miR-130a present in circulating EVs as potential biomarkers for FH [169].

## 6. Thyroid Disorders

The thyroid gland is one of the largest endocrine glands in the body [170], and EVs containing undegraded thyroglobulin (Tg) have been suggested as an alternative way to conventional exocytosis of Tg [170, 171]. The presence of Tg-containing EVs in bovine serum [172] suggests the physiological involvement of this mechanism in the hormone release into circulation. In pathophysiological conditions, such as subclinical hypothyroidism, an increased number of preapoptotic vesicles might promote endothelial dysfunction and cardiovascular risk [173]. Although the precise mechanisms of autoimmunity are still under investigation [174], it is well known that EVs can modulate immune response [175]. Recent studies highlight the importance of an equilibrium between T effector and T regulatory cells to preserve the immune tolerance in thyroid, and of its alterations in the development of the autoimmune thyroid disease (AITD) [176]. Increasing evidence suggests a role of EVs also in thyroid autoimmune disorders. Graves' disease (GD) patients have higher levels of EVs in blood that are significantly reduced after antithyroid therapy with thiamazole [177]. The increase of circulating EVs most likely reflects the activation of immune and inflammatory processes and the resulting cell apoptosis. In addition, patients with GD have higher number of both E-selectin and VE-cadherin-positive EVs, suggesting endothelial dysfunction. The increase in monocyte-EVs indicates an activation and an increased turnover of monocytes. Based on these findings, EVs have been candidate biomarkers for diagnosis and prognosis of GD [177]. In human AITD, EVs seem to have a relevant role in the modulation of the inflammatory response since circulating EVs regulate Tregs and Th17 differentiation [178]. In particular, an increase in platelet-derived EVs and a decrease in leukocyte and endothelial cell-derived EVs have been detected in AITD patients compared to healthy controls. Of note, patient-derived EVs inhibited the *in vitro* differentiation of T regulatory cells and induced the differentiation of proinflammatory Th17 cells. RNA dysregulation, including both miRNAs and lncRNAs, has been associated to the pathogenesis of AITD [176, 179–181]. EVs derived from AITD patients showed a higher expression of miR-146a and miR-155, involved in the differentiation and function of innate and adaptive immunity [178]. Both of these miRNAs are instrumental for function and development of Treg cells. miR-155 also favors the development of inflammatory T cells including Th17 cells. miR-146a and miR-155 can induce immune cell unbalance characteristic of AITD patients by targeting the SMAD family member 4 (SMAD4) [178]. Circulating EVs may mediate the crosstalk between immune cells, leading to the promotion of cytokine expression in peripheral blood mononuclear cells (PBMCs) and contributing to GD pathogenesis [182]. In particular, four miRNAs (miR-92a-3p, miR-23b-5p, miR-339-5p, and let7g-3p) have been detected in EVs [183], suggesting their role in the upregulated cytokine production in intractable GD. The coincubation of EVs isolated from GD patients' sera can stimulate the mRNA expression of IL-1*β* and TNF-*α* in PBMCs, compared with EVs from GD patients in remission or from healthy controls [182]. GD patients in remission show an increase in circulating miR-23b-5p and miR-92a-3p and a decrease of let-7g-3p and miR-339-5p compared to intractable GD patients [182]. The higher expression of these two miRNAs suggests their role as regulators of immune suppression in proinflammatory mechanisms of autoimmune disorders and their association to disease's remission. In addition, miR-339-5p is decreased in GD patients in remission versus intractable GD patients and regulates the expression of sodium-iodine symporter. This protein is involved in the response to radioactive iodine (RAI) therapy [184], a treatment for hyperactive thyroid states including thyroid cancer and Graves' disease [185]. Together, these studies candidate EVs as accessible biomarkers for monitoring or predicting disease activity as well as therapy outcomes in thyroid diseases.

## 7. The Role of EVs in Preeclampsia

EVs and their RNA cargo have been intensively studied for their role in physiology and pathophysiology in the context of reproduction such as sperm maturation, ovarian follicle and oocyte maturation, as well as fertilization, and pregnancy (as previously reviewed [186, 187]). During pregnancy, placental EVs physiologically increase their concentration into circulation and mediate several biological processes, including endometrium remodeling and immunological communication between the mother and the fetus [188–192]. For instance, hypoxia during early pregnancy induces the release from cytotrophoblast cells and placental MSCs of a higher number of EVs with different contents and activities [193, 194]. EVs have been reported to contribute to spiral artery remodeling essential to provide a satisfactory nutrient exchange at the maternal-fetal interface [195]. One example of EV immunological properties is represented by their capacity to confer viral resistance. In fact, both placenta and trophoblast-derived EVs are able to induce resistance of recipient cells against viral infections [196, 197]. Delorme-Axford et al. [196] demonstrated that this mechanism is mediated by the transfer of specific placental miRNAs. Here, we focus on preeclampsia (PE) and the purported role of EVs. Preeclampsia is a multisystem pregnancy disorder that is associated with major maternal and neonatal morbidities [198, 199]. The cellular mechanisms triggering the development and the progression of PE are still not completely elucidated [200]. PE is characterized by hypertension and a decreased utero-placental blood flow linked with impaired trophoblast invasion. Moreover, hypoxia of the placenta promotes the release of harmful substances into the maternal and feto-placental circulation leading to endothelial dysfunction [201]. Increasing evidence suggests that EVs contribute to the initiation and progression of PE by mediating the complex interactions at the maternal-fetal interface [192, 200–202]. In normal pregnancy, EVs released from the syncytiotrophoblast (STB) (STB-EVs) contribute to communication between the maternal endometrium and the embryo [203]. In PE, STB-EVs are released in significantly increased number and show proinflammatory, procoagulant, and antiangiogenic activities, implicating them in the maternal systemic inflammation and endothelial dysfunction [204, 205]. EVs promote vasculogenesis and angiogenesis possibly by transferring miRNAs [201]. Placental EVs contain placental-specific molecules, including proteins (e.g., placental alkaline phosphatase (PLAP)) and miRNAs (e.g., chromosome 19 miRNA cluster) [206, 207]. EVs can also transfer placental-specific miRNA (miR-571a-3p) as it has been shown into human Treg cells and Jurkat leukemic T-cell line, inducing the repression of a gene target (PRKG1) [208]. Increasing studies have highlighted the potential use of EVs and placenta-derived miRNAs as PE diagnostic tools [192, 205, 209, 210]. In fact, EVs are increased in PE maternal blood [191] and early onset PE [211]. Oxygen tension and hypoxia can modulate the release and the content of EVs from placental and trophoblast cells [193, 194, 212–214]. Using an *in vitro* model of PE, it has been demonstrated an alteration of miRNA cargo in a specific subpopulation of STB-EVs, with a downregulation of miR-517a, miR-517b, and miR-141 [215]. In a recent study, Salomon et al. [216] demonstrated an increase of total and placenta-EVs in plasma of PE pregnancies compared with healthy subjects. Importantly, EVs in PE patients showed a different content of miRNAs, mainly related to biological processes dysregulated in PE such as migration, placenta development, and angiogenesis. Among miRNAs, the authors identified the upregulation of miR-486-1-5p and miR-486-2-5p as candidate biomarkers to distinguish PE and normal pregnancies for the early detection of women at risk [216]. Therefore, it is plausible that EVs and their miRNA cargo could be useful as early biomarkers of PE thus improving pregnancy outcomes through the prevention and the reduction of PE severity. In the future, more information about the role of EVs and their associated RNAs will provide a better understanding as to their capacity to modulate gene targets, endothelial dysfunction, and angiogenesis.

## 8. Conclusion

In conclusion, this review summarizes the current knowledge on the role of noncoding RNAs shuttled by EVs in endocrine diseases. EVs may act locally as paracrine mediators and/or at distant sites being released in biological fluids. RNAs contained in EVs are described to be essential mediators of cell communication in endocrine physiopathology. These molecules carried by EVs can modulate the gene expression and the biological function of target cells. The majority of studies have focused on the role of miRNAs carried by EVs since small RNAs are the RNA species more abundant in EVs [205]. Although investigation on EV-lncRNAs and other noncoding RNAs is relatively at the beginning, the evidence suggests their key role in association to miRNAs in regulation of the endocrine system.

EVs and their associated noncoding RNAs are involved in the physiological cellular communication in endocrine organs. In the pancreas, EVs mediate the exchange of information between beta cells with endothelial cells and adjacent beta cells [129, 130]. Through their RNA cargo, EVs regulate beta cell function, insulin secretion, and angiogenesis [131]. In the liver, they can control the metabolic homeostasis and modulate glucose and lipid metabolism [119, 120]. EVs are also essential mediators of normal communication in endocrine glands, such as thyroid, and in the immune system [171, 172, 175, 217]. EV modulation of immune response and angiogenesis suggests also their involvement in reproductive processes. In particular, during pregnancy, EVs and their cargo mediate the immunological interaction at the maternal-fetus interface and control endothelial remodeling [188–192, 195]. Moreover, RNAs carried by EVs have been involved also in the establishment and progression of several endocrine diseases, such as thyroid autoimmunity disorders, complicated pregnancy, and in metabolic dysfunctions, including obesity and diabetes, and their related manifestations. In metabolic syndrome, EV-associated noncoding RNAs can modulate biological processes (oxidative stress, inflammation, insulin signaling, adipogenesis, and angiogenesis) which can promote disease progression. EVs released by macrophages of adipose tissue can modulate liver and muscle glucose and insulin metabolism, favoring diabetes [103]. Skeletal muscle can also modulate the fate of pancreatic beta cells through EV-RNAs leading to insulin resistance [143]. In addition, adipocytes can release EVs that can activate macrophages and lead to inflammation and insulin resistance in obese subjects [105]. EVs from adipose tissue can mediate liver insults and promote chronic inflammation [109, 127] and thus favor the atherosclerosis progression [157, 158]. The modulatory role of EVs in immune response and inflammation has been reported in autoimmune endocrine disorders [182] and in preeclampsia [204, 205]. A better understanding of the role of EVs and their molecular content in pathological and physiological conditions may provide insight for new therapeutic strategies. EVs and their associated noncoding RNAs could be targeted as therapeutic approach. When EV-associated RNAs positively correlate with the progression of endocrine diseases, interventions aimed to reduce their release or their bioactive cargo could inhibit disease. For instance, miR-320 contained in EVs from diabetic rat can inhibit angiogenesis, but a decreased miR-320 content in EVs can restore and have a therapeutic impact [218]. Otherwise, the loading of EVs with therapeutic RNAs can have a beneficial relevance and the enrichment of endothelial cell-derived EVs with miR-146a demonstrated to attenuate dementia-like pathology following administration in diabetic db/db mice [219].

On the other hand, since EVs carry the molecular signature of the cell of origin, they can be exploited as diagnostic tools. In fact, EVs present in the biological fluids vary in number and molecular content depending on the physiological or pathological conditions. In particular, noncoding RNAs present in EVs may provide a picture of ongoing biological processes in the organism. In fact, vesicle-included RNA species are protected from enzyme degradation and are an accessible source of RNAs released in the biological fluid by different cell types and organs. To date, EVs represent a potential diagnostic instrument and increasing studies are highlighting their utility to identify endocrine diseases. In fact, a recent clinical trial has begun to investigate EVs released by beta cells isolated from plasma of diabetes mellitus patients to identify islet-specific antigens (ClinicalTrials.gov identifier: NCT03106246). Likewise, EV-associated noncoding RNAs are arising interest as disease's biomarkers and they are currently investigated in a clinical trial focused on cholangiocarcinoma (ClinicalTrials.gov identifier: NCT03102268). In endocrine diseases, current results on EV-associated noncoding RNAs here presented and summarized in Table 2 give hope to exploit them as an easy and noninvasive diagnostic instrument. Moreover, RNA cargo of EVs could provide clinically useful information and be exploited to staging and in predicting response to therapy. In that context, the optimal definition of the EV isolation procedures is an essential challenge to improve the purity of starting material for downstream analysis. In particular, standardization of techniques will better define the presence of coisolated free circulating RNAs. These non-EV-associated molecules are connected to protein (e.g., Ago2) or lipoprotein complexes, and they can be coisolated with EV-associated RNAs during isolation procedures [55]. Different EV isolation techniques allow different grades of non-EV RNA contamination and can lead to discordant results in the literature. In future, the constant progress and consensus in technology platforms may solve the presence of discrepancies due to technical methodologies which still exist.

## Figures and Tables

**Table 1:** EV-miRNA molecules in endocrine diseases.

Pathology	EV source	miRNA alteration	Target	Recipient cell	Activity	Reference
*Metabolic syndrome*	Macrophages of adipose tissue of obese mice	↑ miR-155	PPAR*γ*	Liver and muscle cells	Glucose metabolism, insulin sensitivity	[103]
Obesity	Obese visceral adipocytes	↓ miR-148b ↓ miR-4269 ↑ miR-23b ↑ miR-4429	TGF-*β* Wnt/*β*-catenin	—	Involvement in liver diseases	[107]
	White and mostly brown adipocytes	miR-99b miR-325 miR-743b miR-98	FGF21 UCP1 PGCI*α*	Liver cells	Metabolic injury (glucose metabolism injury)	[109]
Liver	ASCs of obese subjects	↓ miR-126	↑ Spred1 ↑ E2K1/2 MAPK	—	Reduced EC migration and angiogenesis	[111]
	NAFLD mouse model	↑ miR-122 ↑ miR-192	—	—	EVs as potential biomarkers of hepatic injury	[125]
Pancreas	Human pancreatic islets	miR-27b miR-126 miR-130 miR-296	—	Human islet ECs	Beta cell function, insulin secretion, angiogenesis	[131]
	EPCs	miR-126 miR-296	—	Human islet ECs	Angiogenesis promotion *in vitro* and *in vivo*	[132]
Diabetes	Skeletal muscle	miR-16	Ptch1	Pancreatic beta cells	Development of insulin resistance	[142]
	Urine of patients with diabetic nephropathy	↑ miR-130a ↑ miR-145 ↓ miR-155 ↓ miR-424	—	—	Involvement in diabetes progression	[152]
	ECs	miR-143/miR-145 cluster	—	Vascular smooth muscle cells	Endothelium stabilization	[164]

*Thyroid disorders*	AITD patients	↑ miR-146a ↑ miR-155	SMAD4	—	Function and development of Treg and Th17 cells	[176]
	Intractable Graves' disease	miR-92a-3p miR-23b-5p miR-339-5p let7g-3p	—	—	Potential role in upregulation of cytokine production	[181]

*Preeclampsia*	STB in *in vitro* model of PE	↓ miR-517a ↓ miR-517b ↓ miR-141	—	—	—	[213]
	PE patients	↑ miR-486-1-5p ↑ miR-486-2-5p	—	—	Potential PE biomarkers	[214]

EV: extracellular vesicle; PPAR*γ*: peroxisome proliferator-activated receptor gamma; TGF-*β*: transforming growth factor beta; FGF21: fibroblast growth factor-21; UCP1: uncoupling protein 1; PGCI*α*: peroxisome proliferator-activated receptor gamma coactivator 1-alpha; ASCs: adipose mesenchymal stem cells; Spred1: sprouty-related EVH1 domain-containing 1; E2K1/2 MAPK: extracellular signal-regulated kinase 1/2 mitogen-activated protein kinase; EC: endothelial cell; NAFLD: nonalcoholic fatty liver disease; EPCs: endothelial progenitor cells; Ptch1: protein patched homolog 1; AITD: autoimmune thyroid disease; SMAD4: SMAD family member 4; Treg: T regulatory cells; Th17: T helper 17 cells; STB: syncytiotrophoblast; PE: preeclampsia. Expression trend: ↑: miRNA increased expression; ↓: miRNA reduced expression.

**Table 2:** Most relevant EV-miRNAs for biomarker application in endocrine diseases.

miRNA	Change	Source	Disease		Reference
*miR-23b*	↑	Adipocyte-derived EVs	Obesity	*Metabolic syndrome*	[107]
*miR-122*	↑	Circulating EVs	NAFLD	*Metabolic syndrome*	[125]
*miR-130a*	↑	Urinary EVs	Diabetic nephropathy	*Metabolic syndrome*	[152]
	↓	Circulating EVs	Coronary atherosclerosis	*Metabolic syndrome*	[169]
*miR-141*	↓	STB-derived EVs	Preeclampsia		[215]
*miR-145*	↑	Urinary EVs	Diabetic nephropathy	*Metabolic syndrome*	[152]
*miR-146a*	↑	Circulating EVs	AITD	*Thyroid disorders*	[178]
*miR-148b*	↓	Adipocyte-derived EVs	Obesity	*Metabolic syndrome*	[107]
*miR-155*	↓	Urinary EVs	Diabetic nephropathy	*Metabolic syndrome*	[152]
	↑	Circulating EVs	AITD	*Thyroid disorders*	[178]
*miR-192*	↑	Circulating EVs	NAFLD	*Metabolic syndrome*	[125]
*miR-424*	↓	Urinary EVs	Diabetic nephropathy	*Metabolic syndrome*	[152]
*miR-486-1-5p*	↑	Circulating EVs	Preeclampsia		[216]
*miR-486-2-5p*	↑	Circulating EVs	Preeclampsia		[216]
*miR-517a*	↓	STB-derived EVs	Preeclampsia		[215]
*miR-517b*	↓	STB-derived EVs	Preeclampsia		[215]
*miR-4269*	↓	Adipocyte-derived EVs	Obesity	*Metabolic syndrome*	[107]
*miR-4429*	↑	Adipocyte-derived EVs	Obesity	*Metabolic syndrome*	[107]

EV: extracellular vesicle; NAFLD: nonalcoholic fatty liver disease; STB: syncytiotrophoblast; AITD: autoimmune thyroid disease. miRNA expression change: ↑: increased expression; ↓: reduced expression.

## Abbreviations

AITD: Autoimmune thyroid disease
ALIX: ALG-2-interacting protein X or programmed cell death 6 interacting protein
ALT: Alanine aminotransferase
ARF6: ADP-ribosylation factor 6
ARRDC1: Arrestin domain-containing protein-1
ASCs: Adipose mesenchymal stem cells
CASMC: Coronary artery smooth muscle cells
CDAA: Choline deficient L-amino acid
ceRNAs: Competing endogenous RNAs
cRNAs: Circular RNAs
DM: Diabetes mellitus
EPCs: Endothelial progenitor cells
Erk1/2 MAPK: Extracellular signal-regulated kinase 1/2 mitogen-activated protein kinase
ESCRT: Endosomal sorting complex required for transport
EVs: Extracellular vesicles
FGF21: Fibroblast growth factor-21
FH: Familial hypercholesterolemia
*g*: Centrifugal forces
GD: Graves' disease
hnRNPA2B1: Heterogeneous nuclear ribonucleoprotein A2B1
Ins1: Insulin gene 1
Ins2: Insulin gene 2
IR: Insulin resistance
KLF2: Kruppel-like factor
KLF5: Kruppel-like factor 5
lncRNAs: Long noncoding RNAs
miRNAs: MicroRNAs
Myrip: Myosin VIIA and Rab-interacting protein
MSCs: Mesenchymal stromal cells
MVB: Multivesicular bodies
NAFLD: Nonalcoholic fatty liver disease
NASH: Nonalcoholic steatohepatitis
Pax-6: Paired box protein Pax-6
PBMCs: Peripheral blood mononuclear cells
PE: Preeclampsia
PEG: Polyethylene glycol
PGCI*α*: Peroxisome proliferator-activated receptor gamma coactivator 1-alpha
PM: Plasma membrane
PPAR*γ*: Peroxisome proliferator-activated receptor gamma
RAI: Radioactive iodine
RISC: RNA-induced silencing complex
ROCK1: Rho-associated coiled-coil containing protein kinase 1
rRNAs: Ribosomal RNAs
SEC: Size-exclusion chromatography
siRNAs: Small interference RNAs
SMAD4: SMAD family member 4
snoRNAs: Small nucleolar RNAs
snRNAs: Small nuclear RNAs
STB: Syncytiotrophoblast
T1D: Type 1 diabetes
T2D: Type 2, non-insulin-dependent diabetes
Tg: Thyroglobulin
TGF*β*: Transforming growth factor *β*
TRAIL: Tumor necrosis factor-related apoptosis-inducing ligand
tRNAs: Transfer RNAs
TSG101: Tumour susceptibility gene 101 protein
UCP-1: Uncoupling protein 1
vtRNAs: Vault RNAs
YBX1: RNA-binding protein Y-box protein I
yRNAs: Cytoplasmic Y RNAs.
